# Obesity Incidence According to Branched-Chain Amino Acid Intake and Plant-Based Diet Index Among Brazilian Adults: A Six-Year Follow-Up of the CUME Study

**DOI:** 10.3390/nu17020227

**Published:** 2025-01-09

**Authors:** Fernanda Maria Oliveira da Silva, Adriano Marçal Pimenta, Leidjaira Lopes Juvanhol, Helen Hermana Miranda Hermsdorff, Josefina Bressan

**Affiliations:** 1 Laboratory of Energy Metabolism and Body Composition, Department of Nutrition and Health, Federal University of Viçosa, Viçosa 36570-900, Brazil; fernandanutoli@gmail.com (F.M.O.d.S.); helenhermana@ufv.br (H.H.M.H.); 2 Laboratory of Clinical Analysis and Genomics, Department of Nutrition and Health, Federal University of Viçosa, Viçosa 36570-900, Brazil; 3 Department of Nursing, Federal University of Paraná, Curitiba 80210-170, Brazil; adrianompimenta@gmail.com; 4 Department of Nutrition and Health, Federal University of Viçosa, Viçosa 36570-900, Brazil; leidjaira@ufv.br

**Keywords:** obesity, branched-chain amino acids, plant-based diet

## Abstract

Background: Few studies have evaluated the impact of branched-chain amino acid (BCAA) intake on the risk of obesity in adults. The results are contradictory, and the causality has not been explored. This study assessed the association between BCAA intake and obesity incidence among Brazilian adults and investigated the potential moderating role of the plant-based index (PDI) in this relationship. Methods: A longitudinal study was conducted between 2016 and 2022, with 3090 participants (2043 women, 1047 men; mean age 34 years) from the Cohort of Universities of Minas Gerais (CUME) Study. Data were collected through an online questionnaire. The relationship between BCAA intake and obesity incidence was assessed using crude and adjusted Cox regression models. Restricted cubic spline analysis (RCS) was used to estimate the nonlinearity. The multiplicative interaction with PDI was tested. Results: The overall incidence of obesity was 192 cases (6.21%). The incidence was 16.4/1000 person-years in females; 21.8/1000 person-years in males; and 18.3/1000 person-years total, with a mean follow-up period of 3.4 years. Compared to the first tertile, the highest intake tertiles for BCAA (HR = 1.50, 95% CI = 1.03–2.18), isoleucine (HR = 1.52, 95% CI = 1.04–2.22), and leucine (HR = 1.51, 95% CI = 1.03–2.20) were independently associated with obesity risk. BCAA intake above 16 g/day increases the risk of obesity. Conclusions: There was a positive association between the intake of BCAA, isoleucine, and leucine with the risk of obesity. The PDI accentuated the association between BCAA intake and obesity in both the lowest and highest quintiles.

## 1. Introduction

Obesity is a complex multifactorial disease resulting from the interaction of genetic, environmental, psychosocial, and lifestyle factors [1]. In Brazil, the National Health Survey [2] showed an obesity prevalence of 25.9% in individuals aged over 18 years. Obesity is a major risk factor for other chronic noncommunicable diseases (NCDs), such as cardiovascular diseases, neoplasms, and diabetes, which together are responsible for nearly 30 million deaths annually [3]. Studies have investigated the role of diet and food composition in the genesis of NCDs [4,5]. In recent years, major efforts have been dedicated to investigating how macronutrient composition and diet quality [6] influence chronic conditions, and the beneficial or adverse effects of protein quality and amino acid profile [5,7].

Branched-chain amino acids (BCAAs), namely valine, isoleucine, and leucine, are essential amino acids that cannot be synthesized endogenously and, therefore, must be obtained from animal and plant sources [8]. BCAAs modulate neurotransmission, regulate glucose metabolism, enhance healing processes, improve muscle protein balance in older individuals, and exert beneficial effects in liver and kidney diseases [9].

With the advent of metabolomics, studies were able to identify associations between increased serum BCAA levels and cardiometabolic outcomes, such as cardiovascular risk [10], cardiovascular disease [11], metabolic syndrome [12], and type 2 diabetes mellitus (T2DM) [13]. The relationship between BCAA and obesity, however, has been little explored. McCormack et al. [14], in a cohort study with children and adolescents, found that increased serum BCAA concentrations were associated with increased body mass index (BMI) and a predictor of insulin resistance. In a recent prospective study, Chen et al. [15] reported that higher concentrations of isoleucine and valine were associated with higher homeostatic model assessment for insulin resistance (HOMA-IR) values in overweight adults, but not underweight or normal-weight individuals.

Despite these important associations, few studies have evaluated the impact of BCAA intake on obesity prevalence and risk in adults. Furthermore, some of the existing studies reported contradictory findings. Whereas Okekunle et al. [16] found negative associations between leucine, isoleucine, and valine intake and the likelihood of obesity in the Chinese population, Asoudeh et al. [17] identified positive associations in the Iranian population. In Brazil, Cocate et al. [18] found a lower prevalence of central obesity in the highest tertile of leucine intake.

Furthermore, whether the intake of branched-chain amino acids increases their fasting levels is not established. A study showed that the risk of hypertension increased when the consumption of leucine, valine, and isoleucine exceeded a certain range, in a nonlinear relationship, which makes it important to investigate this intake level on obesity outcomes [19].

Given that nutrients are not consumed isolated, but rather integrated, into a dietary pattern, new approaches have been proposed for global dietary assessment. Dietary patterns may influence the BCAA content of the diet and its relationship with health outcomes [20]. In the current context of increasing consumption of plant-based foods, the plant-based diet index (PDI) has been proposed to identify healthful and unhealthful food intake patterns [21,22]. The overall plant-based diet index (PDI) assigns positive scores to plant foods and reverse scores to animal foods, while the healthful plant-based diet index (hPDI) gives positive scores to healthy plant foods (e.g., whole grains, fruits, vegetables) and reverse scores to less healthy plant foods and animal foods. In contrast, the unhealthful plant-based diet index (uPDI) assigns positive scores to less healthy plant foods and reverse scores to healthy plant foods and animal foods [21].

A meta-analysis of prospective studies showed that the plant-based diet index (PDI) had a protective association with body weight gain and adiposity. Some factors attributed to the lower risk include its lower calorie density, high fiber content, and potential to modulate the gut microbiota and systemic inflammation [23].

In view of the foregoing, this study aimed to prospectively assess the impact of BCAA intake on obesity incidence in the Brazilian adult population and investigate the potential moderating role of PDI in this relationship.

## 2. Materials and Methods

### 2.1. Study Design

The Cohort of Universities of Minas Gerais (CUME) is an observational, open, concurrent, epidemiological study, with a defined population group. The project has been conducted in Brazil since 2016, with students attending seven federal public institutions of higher education in Minas Gerais State. The objective is to assess the impact of the Brazilian dietary pattern and nutritional transition on NCDs.

The CUME study is governed by the ethical principles of non-maleficence, beneficence, justice, and autonomy described in Resolution No. 466/12 of the Brazilian National Health Council and was approved by participating institutions’ Human Research Ethics Committee (protocol No. 3.909.907).

Initially, to carry out the study, invitations were sent to the email addresses of individuals who met the inclusion and eligibility criteria (being over 18 years old, being a native Brazilian, residing in Brazil, and being a graduate of the federal institutions participating in the research). Refusals were considered when, after sending five invitations, no response was received.

Participants were recruited every two years, resulting in continuous sample growth with each follow-up wave. Previously recruited participants received new questionnaires (Q_2, Q_4, …, Q_*n*), and new participants received the baseline questionnaire (Q_0). Follow-up questionnaires contained questions regarding changes in lifestyle, dietary habits, health conditions, and disease incidence. The study design, dissemination strategies, and profile of participants at the first baseline are described in detail in a previous publication [24].

### 2.2. Study Population

To evaluate the association between BCAA consumption and its impact on the incidence of obesity, longitudinal data on the accumulated incidence were used from the Q_0, Q_2, Q_4, and Q_6 questionnaires applied in 2016, 2018, 2020, and 2022, taking as a reference the food consumption questionnaire Q_0.

**Figure 1:**
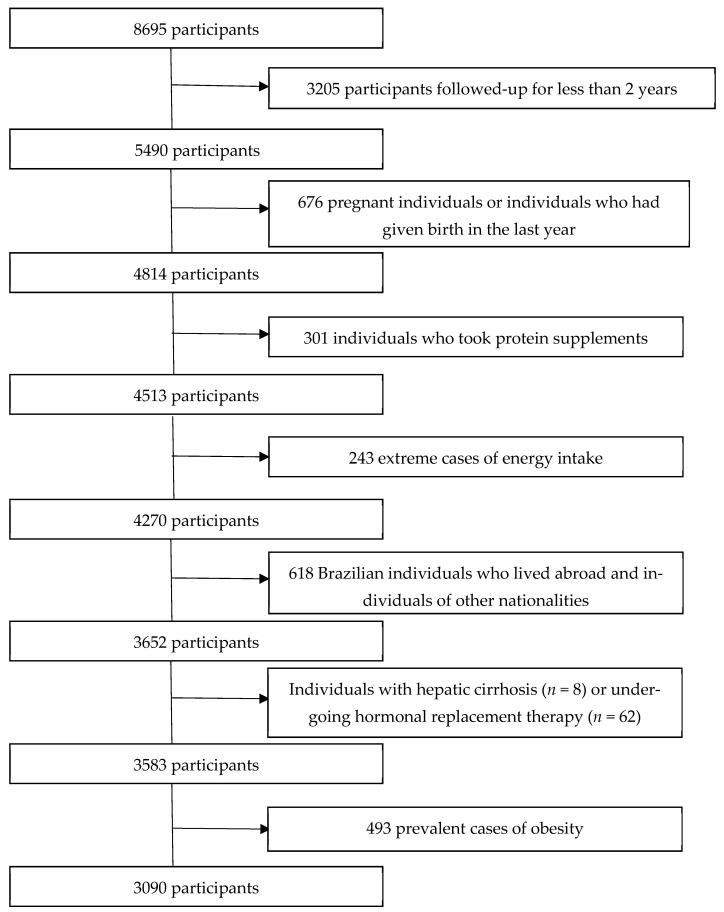
Flowchart of the participant selection process, CUME project (2016–2022).

Between 2016 and 2022, 8695 participants responded to the baseline questionnaire (Q_0). Exclusion criteria were participants without follow-up data (*n* = 3205), of other nationalities (*n* = 53), Brazilians living abroad (*n* = 565), individuals with liver cirrhosis (*n* = 7), extremes of energy intake (<500 kcal/day or >6000 kcal/day) (*n* = 243), pregnant individuals and those who had given birth between baseline and follow-up (*n* = 676), individuals who used protein supplements (*n* = 301), individuals undergoing hormone replacement therapy (*n* = 62), and prevalent cases of obesity (*n* = 493). Thus, the final sample comprised 3090 participants (Figure 1).

### 2.3. Data Collection

Data collection was carried out using online questionnaires. The baseline questionnaire (Q_0) contained two sections, which were sent separately to participants with a 1-week interval. The first section consisted of 83 questions related to sociodemographic information, behavioral characteristics, self-reported biochemical tests, use of medications, previous diagnoses, and individual and family history of diseases. The second section was a quantitative food consumption frequency questionnaire (FCFQ) composed of 144 items and validated for the Brazilian population [25]. A follow-up questionnaire (Q_2, Q_4, and Q_6) was administered every two years. This procedure allowed us to examine the incidence of chronic conditions and changes in behavioral habits compared with baseline (Q_0).

### 2.4. Outcome Variable

In this study, the dependent variable was obesity. Self-reported weight (kg) and height (m) data were used to calculate BMI values, which were previously validated by Miranda et al. (2017). The BMI was calculated by dividing body weight (kg) by height squared (m^2^). Participants with BMI ≥ 30 kg/m^2^ at follow-up but not at baseline were classified as incident cases of obesity [26] (WHO 1999).

### 2.5. Exposure Variable

The main exposure variable of this study was BCAA intake. For data collection, food items were grouped as follows: dairy products; meat and fish, including sausages and eggs; cereals and legumes; fats and oils; fruits; vegetables; beverages (beer and distilled alcoholic beverages, soft drinks, and natural and industrial juices); and other foods (snacks, sweet desserts, salty snacks, sweeteners, sugars, and salt). The consumption frequency of a given food within the previous year was classified as daily, monthly, weekly, or annual. Participants were also asked to indicate portion sizes. Images of food items and utensils were provided to assist participants in selecting food type and portion size, expressed in household measurements or traditional portions.

Consumption frequencies were transformed into daily consumption for each food item. The daily food intake (grams or milliliters) was calculated by multiplying the portion size by the consumption frequency. Information obtained from the FCFQ was used to estimate the intake of energy, macronutrients, (carbohydrates, proteins, and lipids), leucine, isoleucine, valine, and total BCAA. Food consumption variables were adjusted for total energy intake by the residual method [27].

Estimation of food consumption and macronutrient and micronutrient contents of food was carried out using Dietpro^®^ software version 5i. The software provides the Brazilian food chemical composition table [28] and the USDA food chemical composition table. Both tables provide the composition of amino acids per serving (100 g), which was taken as the basis for calculating the portion size of the food item. The Brazilian food composition table [28] was used to compare the nutritional composition of foods that are typical Brazilian dishes and preparations that were adapted to the USDA table, when not available in the Brazilian table. BCAA intake was expressed in grams and classified in tertiles.

### 2.6. Covariates

#### 2.6.1. PDI

Overall PDI, healthful PDI (hPDI), and unhealthful PDI (uPDI) were determined using pre-defined scores [21,22]. The 18 food groups were classified into three major categories, namely healthful plant foods (*n* = 7) (whole grains, fruits, vegetables, nuts, legumes, vegetable oils, tea/coffee), less healthful plant foods (*n* = 5) (fruit juice, refined grains, potatoes, sugary drinks, sweets/desserts), and animal source foods (*n* = 6) (animal fat including butter or lard, dairy products, eggs, fish/shellfish, meat, other animal source foods). Categorization of healthful and less healthful plant foods was based on nutrient similarities, culinary use, and food associations with T2DM, cardiovascular disease, cancer, obesity, high blood pressure, hyperlipidemia, and inflammation [21,22]. Consumption of the 18 food groups (servings/day) was categorized into quintiles (Q1–Q5) and received positive or reverse scores ranging from 1 to 5. For positive scoring, participants classified in the highest quintile of a food group received a score of 5 (e.g., Q1 = 1, Q2 = 2, Q3 = 3, Q4 = 4, and Q5 = 5), whereas, for reverse scoring, the scoring system was reversed (e.g., Q5 = 1, Q4 = 2, Q3 = 3, Q2 = 4, Q1 = 5).

For the classification of overall PDI, healthful and less healthful plant food groups received positive scores, whereas the animal source food group received reverse scores. For hPDI scoring, positive scores were assigned to the healthful plant food group and reverse scores to less healthful plant food and animal source food groups. Finally, for uPDI scoring, positive scores were assigned to the less healthful plant food group and reverse scores to the healthful plant food and animal source food groups. The scores of the 18 food groups were added to calculate overall PDI, hPDI, and uPDI, ranging from 18 to 90. The highest score reflects the lowest intake of animal source foods. For analysis purposes, the scores for overall PDI, hPDI, and uPDI were classified into quintiles.

#### 2.6.2. Sociodemographic Variables

The following sociodemographic variables were determined using the baseline questionnaire: age group (18–29, 30–39, 40–49, 50–59, and ≥60 years), sex (female, male), marital status (married/common-law, divorced/separated, single, widowed/other), level of education (doctoral/postdoctoral degree, master’s degree, specialization, undergraduate degree), per capita income (household income divided by the number of individuals in the household), and employment status (retired/homemaker, unemployed, student, full-time, part-time).

#### 2.6.3. Behavioral Variables

Binge drinking was defined as consuming ≥4 doses of alcoholic beverages for women or ≥5 doses for men on a single occasion in the last 30 days [29]. Physical activity level was determined by the mean number of days and duration (min) of physical activity practice per week. Intensity was determined using a subjective scale (ranging from 0–5 to 10). A list was provided with 23 types of physical activities and sports, with duration expressed in minutes or hours [30]. Individuals who practiced ≥150 min/week of moderate-intensity activity or ≥75 min/week of vigorous-intensity activity were considered active. Physical inactivity was defined as not complying to the minimum amount of physical activity [31].

The usual daily sleep duration and daily time spent using a computer were measured by the questions “In the last 12 months, how much time on average have you spent (1) sleeping at night and (2) using the computer per day (in hours)?” The results were categorized as <7 h or ≥7 h for sleep and <8 h or ≥8 h for computer use.

### 2.7. Statistical Analysis

Participant characteristics were expressed as absolute and relative frequencies for categorical variables and median and interquartile range for quantitative variables. Statistical differences were assessed using Pearson’s chi-squared test (categorical variables) and Mann–Whitney or Kruskal–Wallis tests (quantitative variables). The relative contribution (%) of foods to daily BCAA intake was determined by calculating the ratio of individual BCAA content of the food item to the total amount of BCAA provided by all foods, multiplied by 100 [32].

Crude Cox regression models and models adjusted for potential confounders were estimated to assess the association between BCAA intake and obesity incidence. Follow-up time was calculated in person-years for each participant, as follows: the difference between the completion date of the follow-up questionnaire in which obesity incidence was identified and the completion date of the baseline questionnaire; or the difference between the completion date of the last follow-up questionnaire and the completion date of the baseline questionnaire when the outcome was not identified. The adjustment variables were defined using a directed acyclic graph (Figure S1). The first model was adjusted for sex and age, the second model was additionally adjusted for marital status and per capita income, and the third model was adjusted for the previous variables and physical activity, binge drinking, smoking, computer use, daily sleep duration, saturated fat, and overall PDI. The final model (model 3) included a multiplicative interaction term to test whether PDI modifies the relationship between BCAA intake and obesity incidence. Due to limited statistical power for testing interaction effects, we considered an interaction term between intake of BCAA and PDI as statistically significant if *p* < 0.10 [33,34,35]. If significant, stratified analyses were carried out.

To find out the nonlinear relationship between BCAAs and obesity, restricted cubic splines (RCS) analyses were performed. Three knots at the 10th, 50th, and 90th centiles of BCAA were used and the median values were set as the reference. RCS analyses were performed and adjusted for confounders according to model 3 above.

Finally, the independent association of PDI with obesity incidence was tested using crude and adjusted Cox regression models. The association was measured using the hazard ratio (HR) and the respective 95% confidence intervals (CIs). Data analyses were conducted using Stata software version 13.1 (https://www.stata.com). The significance level adopted in all analyses was 5%.

## 3. Results

Of the total of 3090 participants, 192 had incident obesity. The incidence was 16.4/1000 person-years in females; 21.8/1000 person-years in males; and 18.3/1000 person-years total. The mean follow-up time was 3.4 years. Table 1 shows the characteristics of the study sample stratified by obesity incidence. Compared with non-obese participants, those with obesity were more likely to be female, single, with per capita income less than five minimum wages, with lower median carbohydrate intake, and with a higher median intake of energy, protein, total BCAA, valine, isoleucine, and leucine (*p* < 0.05).

Table 2 presents participant characteristics according to tertiles of total BCAA intake. Participants in the highest tertile of BCAA intake were more likely to be female, have a per capita income less than five minimum wages, be physically active, have higher total energy, protein, and saturated fat intakes, and have lower carbohydrate intake (*p* < 0.05). The major foods contributing to BCAA intake were chicken meat (16.53%), unprocessed beef cuts (14.76%), dairy products (18.47%), fish (7.96%), and beans/lentils (6.53%) (Table 3).

### 3.1. BCAAs and Incidence of Obesity

The regression analysis results for the association between BCAA intake and obesity incidence are shown in Table 4. In the adjusted model (model 3), the highest tertile of total BCAA intake was associated with a 50% increase in the incidence of obesity when compared to the first tertile, 1.50 (OR:1.50; 95% CI: 1.03–2.18). Similar results were observed for leucine intakes (OR:1.51; 95% CI: 1.03–2.20) and isoleucine 1.52 (1.04–2.22) (OR:1.52; 95% CI: 1.04–2.22) when analyzed independently.

### 3.2. Interaction Between BCAAs and PDI

The interaction analysis demonstrated that PDI moderates the association between BCAA and the incidence of obesity (interaction *p* < 0.10). Contrary behavior was observed for hPDI and uPDI. Results of the analysis stratified by quintiles (Table 5) demonstrated an interaction in the first and fourth quintiles of PDI for BCAA, isoleucine, and leucine. Finally, overall PDI, hPDI, and uPDI were not independently associated with obesity incidence (Table S1).

### 3.3. Restricted Cubic Spline Analysis (RCS)

**Figure 2:**
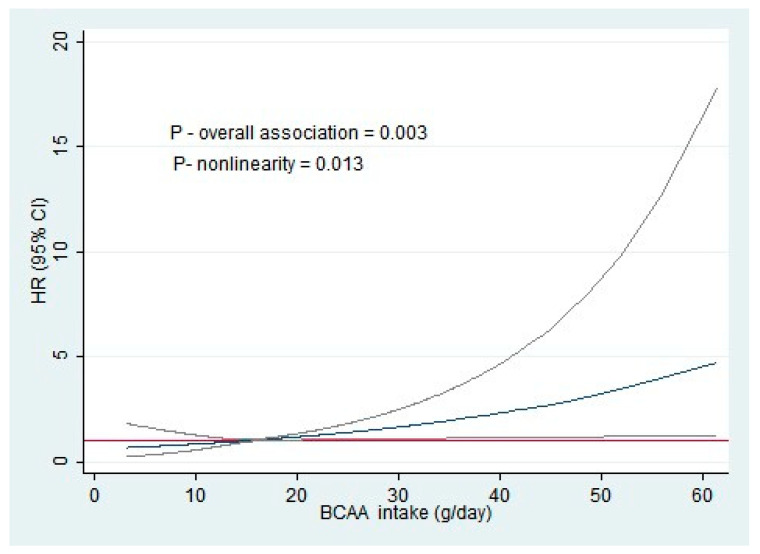
Nonlinear relationship between dietary BCAA intake and obesity risk. The estimate of nonlinearity was adjusted for sex, age, marital status, per capita income, physical activity, binge drinking, smoking status, daily computer time, sleep duration, saturated fat intake, and overall PDI. Note: The light gray lines demonstrate the confidence interval. Abbreviations: BCAA: branched-chain amino acid; HR: hazard ratio; 95% CI: 95% confidence interval.

The nonlinearity analysis performed using RCS models revealed the nonlinear relationships between BCAA intake and obesity. The *p*-value was significant for nonlinearity (*p* = 0.013). When dietary BCAA intake levels exceeded 16 g/day, the risk of obesity increased (Figure 2).

## 4. Discussion

To our knowledge, this is the first prospective study investigating the impact of BCAA intake on the incidence of obesity. We also explored the possible moderating role of plant-based diets in this relationship. The results indicated that the highest tertiles of total intake of BCAA, isoleucine, and leucine were associated with an increased incidence of obesity in the study population. The results of the nonlinearity analysis showed that there is a nonlinear relationship between BCAA intake and the risk of obesity.

Asoudeh et al. found a similar result in the Iranian population; however, various other studies reported an inverse association [16,18,36,37]. Okekunle et al. [38], with diabetes as the outcome variable, and Okekunle et al. [16], with obesity as the outcome variable, found that the highest quartile of BCAA intake was positively and negatively associated with diabetes and obesity, respectively. The magnitude of the association with diabetes was higher for dietary patterns comprising animal source foods, such as milk, meat, and fish than for diets composed of fruits and vegetables. In the cited study, the highest quartile of BCAA intake was inversely associated with obesity, and lower adherence to dietary patterns consisting of sweets, beverages, ice cream, and fish potentiated the inverse association. Here, we evaluated the possible moderating role of PDI in the relationship between BCAA intake and obesity, based on the previously mentioned studies [16,38].

Studies assessing the association between BCAA intake and obesity are scarce and generally of a cross-sectional nature. Furthermore, most did not consider the influence of dietary patterns, possibly contributing to the divergence in results. The direction of the association may depend on the metabolic context and the genetic component of the individual, as the associated mechanisms are not yet completely elucidated.

In line with our findings, cross-sectional studies using metabolomics showed an association between circulating BCAAs and dyslipidemia [13], T2DM [13], and obesity [39]. It is known that 80% of ingested BCAAs reach the bloodstream [40] and that about 50% of isoleucine or leucine and 60% of valine are influenced by diet alone [41]. Higher blood concentrations of BCAAs may reflect an early protein metabolism disorder that may worsen if BCAA intake remains unchanged and/or high [42]. Hamaya et al. demonstrated that women with higher dietary BCAA intake had higher plasma BCAA concentrations in relation to the dose–response, 3.4% higher when comparing the highest with the lowest quintile [43]

In our study, the highest tertile of BCAA intake was associated with the highest obesity incidence. The second and third tertiles of BCAA intake were higher than the levels recommended by Riazi et al. [44], namely 144 mg/kg total BCAA per day. This limit would translate into 10 g of total BCAA per day for a 70 kg man. The study by Asoudeh et al. [17] found an association similar to ours; however, their study did not report the median BCAA consumption. The median consumption of the CUME population was 14.76 g, higher than the median value reported by Okekunle et al. [36] (7.9 g) which obtained a result contrary to ours. It should be noted that Brazilian dietary patterns differ from Philippine dietary patterns. About half of the daily protein intake of Filipino adults stems from cereals, particularly white rice [45]. This suggests that there may be a limit to BCAA intake at which it stops being beneficial. In analyses using RCS models, there was a nonlinear relationship between BCAA consumption and the incidence of obesity. The nonlinear association between dietary BCAA intake and obesity risk had never been reported until the current study. This proves our hypothesis that the risk of obesity could increase when dietary BCAA intake exceeds a suitable range. Our results suggest that there may be a BCAA intake threshold for obesity risk, which may serve as a basis for primary prevention programs. Individuals who take daily BCAA supplements should be warned that there may be adverse effects from excessive BCAA intake.

It is worth noting that in this study, the main sources of BCAAs included unprocessed meats, dairy products, fish, and beans, which are foods widely consumed in the Brazilian diet. Studies on this topic are scarce and most of them involve Asian populations (China and the Philippines). These countries have a different dietary pattern from Brazil, with the predominant consumption of white rice and other cereals in Southeast Asia [36] or a diet with varied protein sources as in China (fish, seafood, tofu, and soy). Therefore, monitoring specific foods that contribute to BCAA intake in different populations may be crucial to better understand the applicability of these findings in other countries.

Furthermore, for a more comprehensive understanding, it would be important to integrate information on other possible dietary controls, such as energy intake and fat intake, consumption of vegetable and animal protein, and dietary patterns in different groups, which could provide more detailed insight into the underlying mechanisms linking BCAA intake to obesity.

Merz et al. [20] demonstrated that plasma BCAA levels can be a mid-term (4 weeks) marker of a diet rich in animal protein (Western standard) and that protein quality is more preponderant than quantity in modulating disease risk. Thus, increased habitual intake of BCAA-rich foods may induce an invariable BCAA state (e.g., deposition in muscle tissue and high turnover rate), with subsequent increase in plasma concentrations.

Here, plant-based diet indices (PDI, hPDI, and uPDI) were not associated with obesity, and the association between increased dietary BCAA intake and higher obesity risk was maintained even after adjusting for PDI. However, there was an interaction between the first and fourth quintile of PDI and BCAA, leucine, and isoleucine. We hypothesized that there would be a difference in the relationship between the consumption of branched-chain amino acids and the incidence of obesity according to PDI levels. As expected, we found this association for the first quintile, however, contrary to our hypothesis, we additionally found this association for the fourth PDI quintile. We believe that the fact that the PDI classification includes less healthy plant-based foods, such as fruit juice, refined grains, potatoes, sugary drinks, and sweets/desserts, may have interfered with this association. Furthermore, more studies are needed to confirm this result, as it may be specific to the dietary pattern of our population.

However, it should be noted that the main sources of BCAA were unprocessed chicken and beef, dairy products, fish, and beans/lentils. A meta-analysis of longitudinal studies indicated that dietary total protein and animal protein levels were associated with an increased risk of T2DM; conversely, plant-based protein was not associated with diabetes risk [46]. A systematic review of three cohort studies [23], evaluating the association between adherence to plant-based dietary patterns and obesity risk, showed an inverse association between hPDI and obesity risk, and a direct association between uPDI and obesity. Healthful plant-based foods are rich in dietary fiber and polyphenols, which increase satiety and have low energy density. These foods also stimulate energy expenditure through thermogenesis and regulation of lipid metabolism, contributing to reducing adiposity. Comparatively, animal source foods that are high in saturated fats, and unhealthful foods that are high in low-quality carbohydrates and added sugars, favor the development of obesity, insulin resistance, and inflammation [23].

A study investigating the relationship between dietary patterns and BCAA content found that a dietary pattern predominantly composed of meats, sausages, sauces, eggs, and ice cream and scarce in oilseeds, cereals, mushrooms, and legumes explained 32.5% of the variation in plasma BCAA. Concomitant with the increase in dietary pattern score, consumption of animal protein was related to a decrease in dietary fiber intake [20].

Multiple pathways associated with BCAAs may contribute to increased obesity risk. Two catabolic enzymes are involved in BCAA metabolism, namely branched-chain amino acid aminotransferase (BCAT) and branched chain α-ketoacid dehydrogenase complex (BCKDH). The latter is regulated by two enzymes, BCKDH kinase (BDK), which inactivates BCKDH, and protein phosphatase 2Cm (PP2Cm), encoded by 1k-dependent protein phosphatase Mg^2+^/Mn^2+^ (PPM1K), responsible for reactivating the complex by dephosphorylation [47].

A recent study identified the binding element carbohydrate response element binding protein (ChREBP), a key transcription factor that regulates sucrose- or fructose-mediated metabolic responses, in the enhancer region of the BDK gene in humans [48]. A diet rich in sucrose or fructose may induce an increase in BDK expression and a decrease in PPM1K expression. Such a diet also activates lipogenic pathways in the liver via pyruvate kinase, ATP citrate lyase, and fatty acid synthase. ATP citrate lyase is related to hepatic steatosis and dyslipidemia [49].

Another hypothesis is that the microbiota of individuals with obesity may increase BCAA production pathways and decrease catabolic pathways. Colonization of germ-free mice with gut microbial communities from obese human individuals led to increased body weight and circulating levels of BCAA and related metabolites, compared with mice colonized with microbiota from lean individuals [50,51]. Excessive exposure to BCAAs can promote excessive lipid storage and impair insulin action through generation of the valine metabolite 3-hydroxybutyrate (3-HIB), which activates transendothelial transport of fatty acids, contributing to tissue lipotoxicity [52].

Clinical studies evaluating the effect of dietary amino acid restriction on metabolic parameters are scarce. Ramzan et al. [53] proposed an intervention with an isocaloric, isoprotein, BCAA-restricted diet that met dietary recommendations for healthy individuals. At the end of the 7-day intervention, total circulating BCAA concentrations decreased significantly by 50% from baseline compared with a control diet. However, the decrease in circulating BCAA did not translate into a significant change in blood glucose, insulin, or HOMA-IR [53]. It should be noted that the cited study had a short duration, requiring medium- to long-term interventions to confirm the effects.

Recent studies in animal (rat) models demonstrated that a BCAA-restricted diet (for all three amino acids) is efficient in improving blood glucose and reducing body fat, provided that equivalent amounts of dietary protein and carbohydrates are consumed [54]. BCAA restriction in obese rats fed a high-fat diet resulted in similar body weight, lean and fat mass, and glucose and insulin levels to the standard diet group [55].

The current study is the first to investigate the impact of BCAA intake on obesity incidence in the context of plant-based diets. The strengths of this study include its prospective design and large sample. Some of the limitations include the absence of plasma BCAA determination, which precludes further assessment of the influence of BCAA intake on body concentration. Data on the dietary intake of BCAAs were collected only once during the follow-up period, and we believe that the BCAA dietary intake of participants did not change substantially over time. Furthermore, our findings are exclusive to the Brazilian population but have the potential to be analyzed by other populations.

## 5. Conclusions

The results showed a positive association between total BCAA, isoleucine, and leucine intakes and the risk of obesity. The PDI accentuated the association between BCAA intake and obesity in both the lowest and highest quintiles. Our results suggest that there may be a BCAA intake threshold for obesity risk, which may serve as a basis for primary prevention programs. Cohort studies and randomized controlled trials that take into account the dietary patterns of individuals from different populations are needed to investigate the potential roles of BCAA on obesity incidence.

## Figures and Tables

**Table 1:** Sociodemographic, lifestyle, and food intake characteristics of study participants according to obesity incidence (CUME study, *n* = 3090, 2016–2022).

Variable	Obesity Incidence ^1^			*p*-Value ^2^
	No	Yes	Total	
Sex				
Female	1929 (94,42)	114 (5.58)	2043 (100)	**0.042**
Male	969 (92.55)	78 (7.45)	1047(100)	
Age group (years)				
18–29	870 (95.08)	45 (4.92)	915 (100)	0.333
30–39	1180 (93.13)	87 (6.87)	1267 (100)	
40–49	535 (92.88)	41 (7.12)	576 (100)	
50–59	238 (94.07)	15 (5.93)	253 (100)	
≥60	75 (94.94)	4 (5.06)	79 (100)	
Marital status				
Married/common-law	1300 (94.27)	79 (5.73)	1379 (100)	**0.006**
Divorced/separated	144 (90.57)	15 (9.43)	159 (100)	
Single	1414 (94.02)	90 (5.98)	1504 (100)	
Widowed/other	40 (83.33)	8 (16.67)	48 (100)	
Level of education				
Undergraduate degree	816 (93.69)	55 (6.31)	871 (100)	
Specialization	629 (93.19)	46 (6.81)	675 (100)	0.871
Master’s degree	868 (94.14)	54 (5.86)	922 (100)	
Doctoral/postdoctoral degree	585 (94.05)	37 (5.95)	622 (100)	
Employment status				
Retired/homemaker	72 (94.74)	4 (5.26)	76 (100)	0.478
Unemployed	197 (96.10)	8 (3.90)	205 (100)	
Student	511 (94.11)	32 (5.89)	543 (100)	
Full-time/part-time	2118 (93.47)	148 (6.53)	2266 (100)	
Per capita income				
<5 minimum wages	1856 (93.08)	138 (6.92)	1994 (100)	
5–9 minimum wages	572 (94.08)	36 (5.92)	608 (100)	
≥10 minimum wages	470 (96.31)	18 (3.69)	488 (100)	**0.028**
Binge drinking				
No	1807 (94.41)	107 (5.59)	1914 (100)	0.067
Yes	1091 (92.77)	85 (7.23)	1176 (100)	
Physical activity level				
Active	1591 (93.61)	108 (6.39)	1689 (100)	
Insufficiently active	700 (92.96)	53 (7.04)	753 (100)	0.197
Inactive	617 (95.22)	31 (4.78)	648 (100)	
Smoking				
Never smoked	2323 (94.05)	147 (5.95)	2470 (100)	
Ex-smoker	334 (93.04)	25 (6.96)	359 (100)	
Smoker	241 (92.34)	20 (7.66)	261 (100)	0.454
≥8 h	1794 (93.83)	118 (6.17)	1912 (100)	
Sleep duration				
<7 h	38 (92.68)	3 (7.32)	41 (100)	0.768
≥7 h	2860 (98.80)	189 (6.20)	3049 (100)	
Energy intake (kcal)	2169.37 (1680.01–2768.30)	2373.26 (1835.46–3277.28)	2180.63 (1693.99–2796.27)	**0.003**
Smoking				
Carbohydrate (g)	247.98 (212.28–281.61)	242.37 (197.46–274.63)	247.55 (211.60–281.35)	**0.029**
Protein (g)	93.65 (79.61–109.56)	97.30 (83.83–112.62)	93.85 (79.79–109.68)	**0.031**
Saturated fat (g)	30.66 (25.48–36.04)	31.23 (25.11–37.71)	30.73 (25.45–36.12)	0.166
BCAAs (g) ^3^	14.71 (12.19–17.45)	15.36 (12.86–18.36)	14.76 (12.22–17.51)	**0.017**
Valine (g)	4.19 (3.47–5.01)	4.42 (3.63–5.24)	4.20 (3.48–5.02)	**0.023**
Isoleucine (g)	3.75 (3.09–4.51)	3.90 (3.29–4.71)	3.76 (3.10–4.53)	**0.010**
Leucine (g)	6.64 (5.51–7.92)	6.89 (5.82–8.27)	6.67 (5.53–7.95)	**0.017**
PDI ^3^	54 (50–59)	54 (49–59)	54 (49–59)	0.429
hPDI ^3^	54 (49–59)	54 (49–59)	54 (49–59)	0.702
uPDI ^3^	54 (49–59)	54 (49–59)	54 (49–59)	0.412

^1^ Results are presented as absolute and relative frequencies or median and interquartile range. ^2^ Pearson’s chi-squared test (categorical variables) or Mann–Whitney test (quantitative variables). ^3^ BCAA, branched-chain amino acids; PDI, plant-based diet index; hPDI, healthful plant-based diet index; uPDI, unhealthful plant-based diet index.

**Table 2:** Sociodemographic, lifestyle, and food intake characteristics of study participants according to branched-chain amino acid intake (CUME study, *n* = 3090, 2016–2022).

Variable	BCAA Intake Tertiles ^1^			*p*-Value ^2^
	T1 (<13.17 g)	T2 (13.17–16.42 g)	T3 (>16.42 g)	
Sex				
Female	706 (34.56)	651 (31.86)	686 (33.58)	**0.040**
Male	324 (30.95)	379 (36.20)	344 (32.86)	
Age group (years)				
18–29	295 (32.24)	295 (32.24)	325 (35.52)	
30–39	411 (32.44)	437 (34.49)	419 (33.07)	0.237
40–49	203 (35.24)	197 (34.20)	176 (30.56)	
50–59	89 (35.18)	84 (33.20)	80 (31.62)	
≥60	32 (40.51)	17 (21.52)	30 (37.97)	
Marital status				
Married/common-law	458 (33.21)	487 (35.32)	434 (31.47)	0.086
Divorced/separated	51 (32.08)	45 (28.30)	63 (39.32)	
Single	499 (33.18)	487 (32.38)	518 (34.44)	
Widowed/other	22 (45.83)	11 (22.92)	15 (31.25)	
Level of education				
Undergraduate degree	303 (34.79)	294 (33.75)	274 (31.46)	0.181
Specialization	246 (36.44)	207 (30.67)	222 (32.89)	
Master’s degree	290 (31.45)	309 (33.51)	323 (35.03)	
Doctoral/postdoctoral degree	191 (30.71)	220 (35.37)	211 (33.92)	
Employment status				
Retired/homemaker	31 (40.79)	20 (26.32)	25 (32.89)	0.612
Unemployed	71 (34.63)	65 (31.71)	69 (33.66)	
Student	185 (34.07)	170 (31.31)	188 (34.62)	
Full-time/part-time	743 (32.79)	775 (34.20)	748 (33.01)	
Per capita income				
<5 minimum wages	681 (34.15)	687 (34.45)	626 (31.39)	
5–9 minimum wages	190 (31.25)	191 (31.41)	227 (37.34)	
≥10 minimum wages	159 (32.58)	152 (31.15)	177 (36.27)	**0.043**
Binge drinking				
No	644 (33.65)	627 (32.76)	643 (33.59)	0.687
Yes	386 (32.82)	403 (34.27)	387 (32.91)	
Physical activity level				
Active	525 (31.08)	543 (32.15)	621 (36.77)	**0.000**
Insufficiently active	217 (33.49)	221 (34.10)	210 (32.41)	
Inactive	288 (38.25)	266 (35.33)	199 (26.43)	
Smoking				
Never smoked	97 (37.16)	93 (35.63)	71 (27.20)	
Ex-smoker	806 (32.63)	832 (33.68)	832 (33.68)	
Smoker	127 (35.38)	105 (29.25)	127 (35.38)	0.101
Daily computer use				
<8 h	402 (34.13)	394 (33.45)	382 (32.43)	0.659
≥8 h	628 (32.85)	636 (33.26)	648 (33.89)	
Sleep duration				
<7 h	15 (36.59)	8 (19.51)	18 (43.90)	0.142
≥7 h	1015 (33.29)	1022 (33.52)	1012 (33.19)	
Energy intake (kcal)	2072.81(1509.01–2951.44)	2154.46 (1706.90–2722.30)	2254.50 (1829.84–2773.98)	**0.000**
Carbohydrate (g)	265.11 (226.76–302.73)	252.19 (222.46–281.32)	224.86 (193.66–259.0)	**0.000**
Protein (g)	74.72 (65.65–82.04)	94.75 (88.82–101.27)	115.66 (105.94–128.58)	**0.000**
Saturated fat (g)	27.98 (22.82–33.53)	31.44 (26.78–36.80)	32.31 (27.20–37.40)	**0.000**
PDI ^3^	54 (49–59)	55 (49.5–59)	54 (49–59)	0.803
hPDI ^3^	54 (49–59)	53 (49–58)	54 (49–59)	0.734
uPDI ^3^	54 (49–59)	54 (49–58)	54 (49–59)	0.711

^1^ Results are presented as absolute and relative frequencies or median and interquartile range. ^2^ Pearson’s chi-squared test (categorical variables) or Kruskal–Wallis test (quantitative variables). ^3^ BCAA, branched-chain amino acids; PDI, plant-based diet index; hPDI, healthful plant-based diet index; uPDI, unhealthful plant-based diet index.

**Table 3:** Contribution (%) of food items to branched-chain amino acid intake among participants of the CUME study (*n* = 3090).

Food Item	(%)	Food Item	(%)
Unprocessed meat		Cream cheese	1.90
Chicken (with/without skin)	16.53	Ricotta	1.59
Beef	14.76	Semi-skimmed milk	1.02
Other fish	5.23	Eggs	3.58
Sardine/tuna/salmon/cod	2.73	Other	
Pork	1.74	Beans/lentils	6.53
Lamb	0.13	Peanut/walnut/other nuts	3.01
Offal	0.04	White rice	2.66
Processed meat		Soy milk	1.94
Turkey ham	1.64	Pizza	1.83
Sausage	1.25	Whole bread	1.59
Mortadella	0.74	Cheese bread	1.39
Hot dog sausage	0.33	Lasagna	1.01
Bacon	0.27	Pasta	0.88
Smoked meat	0.15	Sliced bread	0.74
Dairy products		Whole rice	0.55
Cheese	8.21	Sweet bread	0.46
Whole milk	3.50	Soy protein	0.11
Skimmed milk	2.25	Oat/granola	0.09
Total			90.38%

**Table 4:** Hazard ratios (HR) and 95% confidence intervals (CIs, in parentheses) for the association between branched-chain amino acid (BCAA) intake tertiles and obesity incidence (CUME study, *n* = 3090, 2016–2022).

Variable	BCAA Intake Tertiles			Trend *p*-Value
	T1 (<13.16 g)	T2 (13.16–16.42 g)	T3 (>16.42 g)	
Total BCAA				
Crude model	1.00	1.32 (0.91–1.90)	1.55 (1.09–2.21) ^1^	**0.015**
Model 1 ^2^	1.00	1.31 (0.90–1.89)	1.57 (1.10–2.24) ^1^	**0.012**
Model 2 ^3^	1.00	1.36 (0.94–1.98)	1.62 (1.12–2.33) ^1^	**0.009**
Model 3 ^4^	1.00	1.27 (0.87–1.84)	1.50 (1.03–2.18) ^1^	**0.034**
Valine				
Crude model	1.00	1.24 (0.86–1.79)	1.51 (1.06–2.14) ^1^	**0.021**
Model 1 ^2^	1.00	1.31 (0.90–1.89)	1.57 (1.10–2.24) ^1^	**0.020**
Model 2 ^3^	1.00	1.30 (0.90–1.88)	1.58 (1.10–2.26) ^1^	**0.013**
Model 3 ^4^	1.00	1.22 (0.83–1.77)	1.42 (0.97–2.06)	0.065
Leucine				
Crude model	1.00	1.30 (0.90–1.87)	1.60 (1.12–2.28) ^1^	**0.009**
Model 1 ^2^	1.00	1.28 (0.89–1.86)	1.62 (1.13–2.31) ^1^	**0.008**
Model 2 ^3^	1.00	1.37 (0.94–1.99)	1.67 (1.16–2.41) ^1^	**0.006**
Model 3 ^4^	1.00	1.27 (0.87–1.86)	1.51 (1.03–2.20) ^1^	**0.031**
Isoleucine				
Crude model	1.00	1.28 (0.89–1.86)	1.62 (1.14–2.31) ^1^	**0.007**
Model 1 ^2^	1.00	1.28 (0.88–1.85)	1.65 (1.16–2.36) ^1^	**0.005**
Model 2 ^3^	1.00	1.41 (0.97–2.06)	1.69 (1.17–2.43) ^1^	**0.005**
Model 3 ^4^	1.00	1.69 (1.17–2.43)	1.52 (1.04–2.22) ^1^	**0.029**

^1^ *p* < 0.05. ^2^ Model 1, adjusted for sex and age. ^3^ Model 2, adjusted for sex, age group, marital status, and per capita income. ^4^ Model 3, adjusted for sex, age, marital status, per capita income, physical activity, binge drinking, smoking status, daily computer time, sleep duration, saturated fat intake, and overall PDI.

**Table 5:** BCAA consumption–plant-based index (PDI) interaction and associations between BCAA consumption and obesity, stratified by categories of PDI (CUME study, *n* = 3090, 2016–2022).

		BCAA		
PDI (Quintiles)	T1	T2	T3	*p*-Value for Interaction
Q1	1.00	3.14 (1.26–7.82) ^1^	3.34 (1.30–8.53) ^1^	**0.013**
Q2	1.00	0.83 (0.30–2.32)	1.90 (0.75–4.80)	0.174
Q3	1.00	0.69 (0.34–1.40)	0.60 (0.26–1.37)	0.229
Q4	1.00	2.36 (0.97–5.76)	3.06 (1.25–7.84) ^1^	**0.014**
Q5	1.00	1.20 (0.43–3.34)	1.93 (0.74–5.00)	0.720
Isoleucine				
Q1	1.00	2.40 (0.98–5.83)	3.15 (1.29–7.69) ^1,2^	**0.012**
Q2	1.00	0.81 (0.29–2.26)	1.91 (0.75–4.86)	0.694
Q3	1.00	0.92 (0.46–1.87)	0.66 (0.28–1.54)	0.834
Q4	1.00	2.53 (0.98–6.55)	3.80 (1.56–9.27) ^1,2^	**0.003**
Q5	1.00	1.22 (0.46–3.26)	1.73 (0.64–4.65)	0.277
Leucine				
Q1	1.00	2.58 (1.06–6.28)	3.26 (1.34–7.97) ^1,2^	**0.009**
Q2	1.00	0.81 (0.29–2.27)	1.91 (0.74–4.91)	0.699
Q3	1.00	0.77 (0.38–1.56)	0.64 (0.28–1.46)	0.479
Q4	1.00	3.10 (1.23–7.82) ^1,2^	3.40 (1.35–8.59) ^1,2^	**0.016**
Q5	1.00	1.05 (0.37–2.93)	2.05 (0.78–5.36)	0.140

^1^ *p* < 0.05. ^2^ Model 3, adjusted for sex, age, marital status, per capita income, physical activity, binge drinking, smoking status, daily computer time, sleep duration, saturated fat intake, and overall PDI.

## Data Availability

The data described in the manuscript will be made available upon request and pending approval.

## Supplementary Materials

The following supporting information can be downloaded at: https://www.mdpi.com/article/10.3390/nu17020227/s1, Figure S1: Acyclic graph depicting the association between branched-chain amino acid (BCAA) intake and obesity; Table S1: Hazard ratios (HRs) and 95% confidence intervals (CIs) for the association of overall (PDI), healthful (hPDI), and unhealthful (uPDI) plant-based diet indices with obesity incidence (CUME study, *n* = 3090, 2016–2022).
