# Supplementary material for: Obesity Incidence According to Branched-Chain Amino Acid Intake and Plant-Based Diet Index Among Brazilian Adults: A Six-Year Follow-Up of the CUME Study
Source: Nutrients. 2025 Jan 9;17(2):227. doi: 10.3390/nu17020227 (PMC11767458; doi:10.3390/nu17020227)
Supplement: Supplementary file 1 [file nutrients-17-00227-s001.zip › nutrients-3388132-supplementary.pdf]

Supplementary Materials

Supplemental Table S1. Hazard ratios (HR) and 95% confidence intervals (CI) for the association of overall (PDI), healthful (hPDI), and unhealthful (uPDI) plant-based diet indices with obesity incidence (CUME study, *n* = 3090, 2016–2022)

Quartile	Crude model HR (95% CI)	Trend *p*-value	Model 1[1] HR (95% CI)	Trend *p*-value	Model 2[2] HR (95% CI)	Trend *p*-value
			**PDI**			
Q1 (26–48)	1 (ref)		1 (ref)		1 (ref)	
Q2 (49–52)	0.60 (0.37–0.98)		0.60 (0.37–0.98)		0.61 (0.37–0.99)	
Q3 (53–56)	0.89 (0.59–1.36)	0.895	0.89 (0.58–1.36)	0.899	0.90 (0.59–1.39)	0.875
Q4 (57–60)	0.94 (0.61–1.44)		0.94 (0.61–1.48)		0.97 (0.63–1.49)	
Q5 (61–78)	0.78 (0.49–1.24)		0.78 (0.49–1.24)		0.74 (0.46–1.19)	
			**hPDI**			
Q1 (33–48)	1 (ref)		1 (ref)		1 (ref)	
Q2 (49–52)	1.22 (0.79–1.87)		1.20 (0.78–1.85)		1.16 (0.75–1.79)	
Q3 (53–56)	1.21 (0.80–1.84)	0.708	1.19 (0.78–1.81)	0.584	1.06 (0.69–1.62)	0.297
Q4 (57–60	0.75 (0.44–1.25)		0.73 (0.43–1.22		0.63 (0.37–1.09)	
Q5 (61–83)	1.07 (0.68–1.68)		1.06 (0.67–1.67)		0.98 (0.62–1.56)	
			**uPDI**			
Q1 (29–48)	1 (ref)		1 (ref)		1 (ref)	
Q2 (49–52)	1.17 (0.76–1.82)		1.19 (0.77–1.85)		1.16 (0.73–1.82)	
Q3 (53–56)	1.09 (0.71–1.67)	0.572	1.10 (0.72–1.68)	0.450	1.15 (0.74–1.78)	0.535
Q4 (57–60)	1.05 (0.67–1.65)		1.06 (0.67–1.66)		1.08 (0.68–1.71)	

Q5 (61–76)	0.84 (0.52–1.34)	0.84 (0.52–1.35)	0.85 (0.52–1.39)

1 Model 1, adjusted for sex and age
2 Model 2, adjusted for sex, age, marital status, per capita income, physical activity, binge drinking, smoking status, daily computer time, sleep duration, and diabetes.

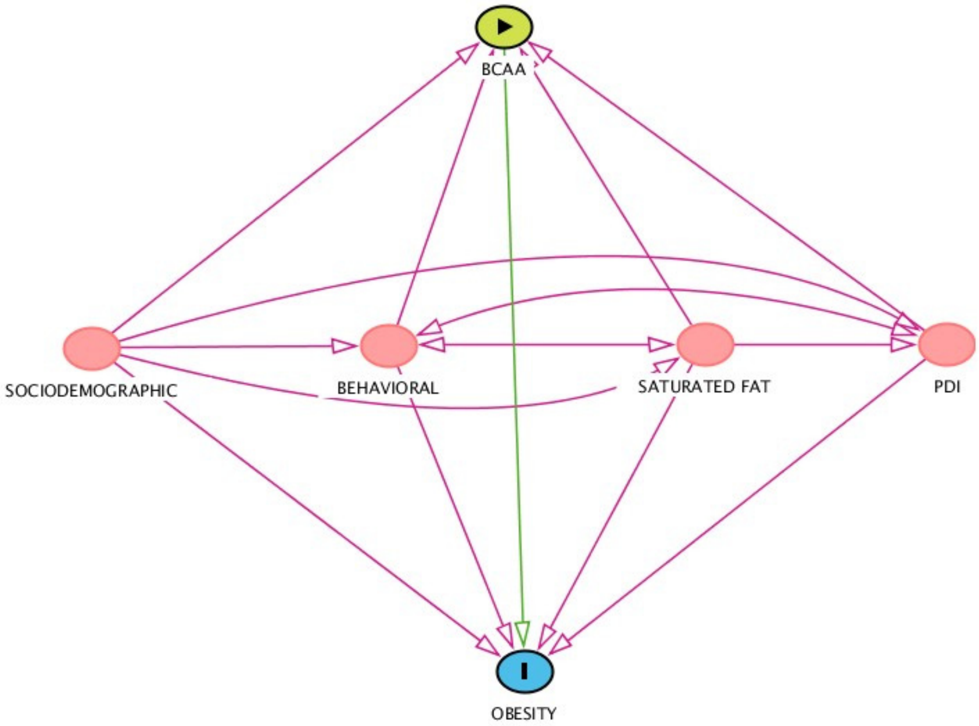

Supplemental Figure S1. Acyclic graph depicting the association between branched-chain amino acid (BCAA) intake and obesity. Obesity is the outcome variable. BCAA (total, valine, leucine, and isoleucine) intake is the exposure variable. Sociodemographic (sex, age, marital status, and per capita income), behavioral (physical activity, smoking, binge drinking, daily computer use, and sleep duration), and food intake (PDI and saturated fat) characteristics are the covariates. PDI, plant-based diet index. Green arrows indicate causality, pink arrows indicate ancestors of the outcome and exposure variables and potential adjustment variables.
